# From Adipose Tissue Dysfunction to Venous Thromboembolism

**DOI:** 10.3390/jcm15197500

**Published:** 2026-09-26

**Authors:** Andrea Boccatonda, Alice Brighenti, Alfredo Caturano, Damiano D’Ardes, Carlo Acierno, Vincenzo Russo, Caterina Conte, Paolo Simioni, Elena Campello

**Affiliations:** 1 Diagnostic and Therapeutic Interventional Ultrasound Unit, IRCCS Azienda Ospedaliero-Universitaria di Bologna, Policlinico Sant’Orsola-Malpighi, Via Massarenti N. 9, 40138 Bologna, Italy; alice.brighenti3@studio.unibo.it; 2 Department of Human Sciences and Promotion of the Quality of Life, San Raffaele Roma University, 00166 Rome, Italy; 3 Institute of “Clinica Medica”, Department of Medicine and Aging Science, G. D’Annunzio University of Chieti-Pescara, 66100 Chieti, Italy; 4 Azienda Ospedaliera Regionale San Carlo, 85100 Potenza, Italy; 5 Division of Cardiology, Department of Medical Translational Sciences, University of Campania Luigi Vanvitelli, 80138 Naples, Italy; 6 Obesity and Obesity-Related Cardiometabolic Complications Research Unit, IRCCS MultiMedica, 20099 Milan, Italy; 7 Internal Medicine, Thrombotic and Hemorrhagic Diseases Unit, Department of Medicine, Padova University Hospital, 35128 Padua, Italyelena.campello@unipd.it (E.C.)

**Keywords:** deep vein thrombosis, pulmonary embolism, GLP-1 receptor agonists, tirzepatide, immunothrombosis, hypercoagulability, weight loss

## Abstract

**Background/Objectives**: Obesity is a major modifiable risk factor for venous thromboembolism (VTE), driven by adipose tissue dysfunction, chronic inflammation, endothelial dysfunction, hypercoagulability, impaired fibrinolysis, immunothrombosis, and venous stasis. Modern incretin-based therapies induce substantial weight loss and improve multiple cardiometabolic abnormalities that may influence thrombotic risk. To critically evaluate the biological rationale and clinical evidence supporting a potential role of incretin-based therapies in modifying VTE risk, while distinguishing mechanistic plausibility from proven clinical benefit and weight-loss-mediated effects from direct pharmacological actions. **Methods**: Narrative review of MEDLINE/PubMed and EMBASE (2000–2026), with critical appraisal of mechanistic studies, randomized trials, pharmacoepidemiological evidence, and current clinical recommendations. **Results**: Experimental and translational studies provide biological plausibility for potential antithrombotic effects of incretin-based therapies through modulation of inflammation, endothelial function, platelet activation, and coagulation pathways; however, these biomarkers are not validated surrogate endpoints for VTE. An expanding body of contemporary observational evidence, including active-comparator studies and target-trial emulations, has generally reported lower rates of VTE, pulmonary embolism, and/or deep vein thrombosis among patients receiving incretin-based therapies, although effect estimates vary according to population, comparator, and clinical setting. In contrast, recent meta-analyses of randomized trials have not demonstrated a significant reduction in overall VTE and have yielded partly conflicting findings for pulmonary embolism and deep vein thrombosis. Major cardiovascular outcome trials were not specifically designed or powered to assess VTE. Current evidence suggests that sustained weight loss is the most plausible mediator of any potential long-term reduction in VTE risk, whereas a clinically relevant weight-independent antithrombotic effect remains unproven. **Conclusions**: Incretin-based therapies have a compelling biological rationale and a growing observational signal for potential VTE risk reduction, but current evidence is insufficient to establish a causal protective effect. Any potential long-term benefit appears most plausibly related to sustained weight loss and reversal of obesity-associated prothrombotic abnormalities, while a clinically relevant weight-independent antithrombotic effect remains unproven. Accordingly, these therapies should continue to be prescribed according to established metabolic indications, and any reduction in VTE risk should be regarded as a potential but unconfirmed additional benefit. Dedicated randomized trials with prespecified and adjudicated VTE outcomes are required before VTE prevention can be considered a therapeutic indication.

## 1. Introduction

In 2022, more than one billion people worldwide were living with obesity, a number that has more than doubled since 1990 and continues to rise [1]. What was once framed primarily as a metabolic predisposition is now recognized as a systemic inflammatory disease, with biological consequences extending well beyond glucose dysregulation and atherosclerosis. Among these consequences, venous thromboembolism (VTE) has become increasingly prominent [2,3]. Adipose tissue dysfunction has emerged as the central biological link between excess adiposity and the inflammatory, endothelial, and haemostatic alterations that ultimately promote venous thrombosis [4,5]. Epidemiological data consistently support an independent, dose-dependent association between body mass index (BMI) and VTE incidence, which is substantially amplified in the presence of concurrent prothrombotic triggers [2,6,7].

Occupational and environmental exposures may also contribute to VTE risk, although the available evidence is less consistent than for established clinical risk factors. Prolonged work- and computer-related seated immobility has been associated with increased VTE risk in case–control studies [8], although subsequent studies have reported weaker or non-significant associations [9]. Consistently, a large prospective study including 78,936 participants found no significant increase in VTE risk among individuals with ≥6.5 h/day of occupational sitting compared with those sitting ≤3.5 h/day (HR 1.11, 95% CI 0.92–1.34), suggesting that sedentary work per se may be less relevant than prolonged uninterrupted immobility [10].

Environmental exposures have also been investigated. Long-term exposure to particulate air pollution has been associated with DVT in observational studies [11], whereas a large Italian nationwide analysis of more than 495,000 hospitalizations found an association between ambient temperature and PE admissions but no consistent association between particulate matter exposure and venous thrombosis [12]. Overall, these findings suggest that occupational immobility and environmental exposures may contribute to thrombotic risk in selected settings, but their effects remain heterogeneous and should be interpreted within the broader context of obesity, physical activity, comorbidities, and individual thrombotic susceptibility. These considerations may be particularly relevant in working-age individuals with obesity, although no evidence currently demonstrates that occupational or environmental exposures modify the effects of incretin-based therapies on VTE risk.

The past decade has also transformed the treatment of obesity. Incretin-based therapies, particularly glucagon-like peptide-1 receptor agonists (GLP-1RAs) and dual glucose-dependent insulinotropic polypeptide/glucagon-like peptide-1 receptor agonists (dual GIP/GLP-1 receptor agonists), produce weight reductions previously achievable only through surgery and confer additional cardiometabolic benefits across multiple organ systems [13,14,15]. Large randomized clinical trials have demonstrated reductions in major adverse cardiovascular events (MACEs) [16,17,18,19], prompting interest in whether similar protection might extend to venous thromboembolism. Despite the growing use of incretin-based therapies, whether their established cardiometabolic benefits translate into meaningful protection against VTE remains uncertain. Moreover, mechanistic insights and emerging clinical evidence have rarely been considered within a unified critical framework [20]. This review critically examines whether the established cardiovascular benefits of incretin-based therapies can reasonably be extended to venous thromboembolism, while avoiding both uncritical extrapolation and excessive scepticism unsupported by current evidence. The arterial and venous thrombotic systems share important substrates, such as inflammation, endothelial dysfunction, and immune thrombosis, but diverge substantially in their dependence on platelet function versus coagulation activation, shear physiology, and triggering mechanisms. These distinctions are important for interpreting existing evidence and designing future studies.

## 2. Literature Search

This narrative review was informed by a structured search of MEDLINE/PubMed and EMBASE for articles published from January 2000 to August 2026. The search combined terms related to obesity and adipose tissue dysfunction with terms related to venous thromboembolism and incretin-based therapies, including “obesity,” “adipose tissue dysfunction,” “venous thromboembolism,” “deep vein thrombosis,” “pulmonary embolism,” “GLP-1 receptor agonists,” “semaglutide,” “tirzepatide,” “coagulation,” “fibrinolysis,” “immunothrombosis,” and “neutrophil extracellular traps.” Boolean operators were adapted to the syntax of each database. Priority was given to systematic reviews, meta-analyses, randomized controlled trials, large prospective or population-based cohort studies, and contemporary clinical guidelines. Mechanistic, translational, and experimental studies were included when they provided relevant evidence for biological pathways not adequately addressed by clinical studies. Reference lists of selected articles were also screened to identify additional relevant publications. Only articles published in English were considered. Study selection was based on relevance to the relationship between obesity, weight loss, incretin-based therapies, and venous thromboembolic risk. Because of the heterogeneity of the available evidence and the broad mechanistic and clinical scope of the topic, no formal meta-analysis or systematic risk-of-bias assessment was performed.

## 3. Obesity and Venous Thromboembolism

### 3.1. Epidemiology

The association between obesity and VTE is one of the most consistently replicated findings in thrombosis epidemiology. The Copenhagen City Heart Study, which included nearly 19,000 participants over a median 19.5-year follow-up, found a hazard ratio of 2.10 for VTE at BMI ≥ 35 kg/m^2^ vs. <20 kg/m^2^ after multivariable adjustment [21]. The MEGA study demonstrated a 2.4-fold increased risk with obesity alone, rising to 24-fold with oral contraceptive use and 7.9-fold with the factor V Leiden mutation [22]. A dose–response meta-analysis of nearly four million participants confirmed a progressive BMI-VTE relationship [23,24]. The population-attributable fraction data illustrate the public health dimension. The Tromsø Study estimated that overweight and obesity account for approximately 25% of all VTE events at the population level [24]. Obesity also predicts recurrence after anticoagulation withdrawal: Eichinger et al. reported an adjusted hazard ratio of 1.6 for recurrence among obese vs. normal-weight patients at four years [25], a finding supported, with heterogeneous results, by a systematic review of 20 studies [26], and consistent with reports that recurrence risk rises incrementally with each additional component of the metabolic syndrome rather than with obesity in isolation [27]. Visceral adiposity appears more pathophysiologically relevant than overall BMI, given its higher metabolic and inflammatory activity; imaging-based body composition measures may ultimately prove superior to BMI for thrombotic risk stratification. Beyond incidence, obesity appears to shape the postoperative VTE presentation and behaves clinically. In the international RIETE registry, the proportion of postoperative VTE presenting as pulmonary embolism rather than isolated DVT increased progressively across BMI categories, among 3196 patients with confirmed postoperative VTE [28]. Paradoxically, patients with obesity showed lower 3-month mortality and major bleeding than normal-weight patients [28]. These findings should be interpreted cautiously because of potential selection bias, residual confounding, differences in comorbidity burden, and the possibility that normal-weight patients developing postoperative VTE represent a more vulnerable subgroup. Although altered leptin signalling has been proposed as a possible contributor to this apparent obesity paradox, the underlying mechanisms remain speculative [27].

### 3.2. Pathophysiology

The mechanistic basis of obesity-associated VTE extends beyond the classical components of Virchow’s triad and originates from adipose tissue dysfunction, which initiates a cascade of inflammatory, metabolic, endothelial, and haemostatic alterations ultimately promoting venous thrombosis [20]. These mechanisms are highly interconnected but can be conceptually divided into adipose tissue dysfunction, hypercoagulability and impaired fibrinolysis, endothelial dysfunction and immune-thrombosis, and venous stasis.

#### 3.2.1. Adipose Tissue Dysfunction

Adipose tissue expansion is accompanied by adipocyte hypertrophy, which exceeds local vascular supply and results in tissue hypoxia. This promotes adipocyte stress, cell death, and the formation of macrophage-rich crown-like structures surrounding dysfunctional adipocytes. Progressive immune-cell infiltration shifts adipose tissue toward a chronic low-grade inflammatory state characterized by sustained activation of innate immunity [29]. Dysfunctional adipose tissue undergoes profound changes in adipokine secretion. Increased leptin and reduced adiponectin contribute to endothelial dysfunction, oxidative stress, platelet activation, and systemic inflammation [30]. Simultaneously, activated macrophages release large amounts of tumor necrosis factor (TNF)-α and interleukin-6 (IL-6), which further amplify inflammatory signalling and stimulate the hepatic production of several procoagulant proteins, thereby reinforcing the hypercoagulable state [31]. Collectively, these alterations transform adipose tissue from a passive energy-storage organ into an active immune-metabolic organ capable of promoting systemic inflammation and creating a prothrombotic milieu.

#### 3.2.2. Hypercoagulability and Impaired Fibrinolysis

The chronic inflammatory environment generated by dysfunctional adipose tissue directly alters the coagulation system. Obesity is associated with increased circulating concentrations of fibrinogen, factors VII and VIII, enhanced tissue factor pathway activation, and elevated von Willebrand factor, collectively promoting thrombin generation and fibrin formation [29,32]. Fibrinolysis is simultaneously impaired. Visceral adipocytes are a major source of plasminogen activator inhibitor-1 (PAI-1), whose expression increases proportionally with adipocyte size [33]. The resulting hypofibrinolytic state promotes clot persistence and likely contributes to an elevated recurrence risk in patients with obesity.

#### 3.2.3. Endothelial Dysfunction and Immuno-Thrombosis

Chronic inflammation also disrupts endothelial homeostasis. The endothelial glycocalyx, which normally limits leukocyte adhesion and preserves nitric oxide bioavailability, is progressively degraded by the inflammatory milieu associated with obesity [34,35]. Loss of glycocalyx integrity is accompanied by reduced nitric oxide production, increased expression of vascular cell adhesion molecule 1 (VCAM-1), intercellular adhesion molecule 1 (ICAM-1), and tissue factor, and enhanced leukocyte-endothelium interactions, collectively generating a prothrombotic endothelial phenotype [29]. Endothelial dysfunction is further amplified by immune-thrombotic mechanisms. Neutrophils infiltrating dysfunctional adipose tissue release neutrophil extracellular traps (NETs), extracellular DNA-protein scaffolds containing citrullinated histones that promote platelet activation, activate coagulation, and stabilize fibrin-rich thrombi [36,37]. Observations during the COVID-19 pandemic further highlighted how obesity amplifies inflammation-driven immune-thrombosis during severe systemic illness [38].

#### 3.2.4. Venous Stasis

In addition to biological alterations, obesity promotes the mechanical component of Virchow’s triad. Increased intra-abdominal pressure impairs venous return from the lower limbs, while reduced mobility, impaired calf-muscle pump function, and chronic venous insufficiency prolong venous blood residence time [39,40,41]. During hospitalization or after surgery, these baseline abnormalities are further exacerbated by prolonged immobilization and acute inflammation, creating optimal conditions for thrombus initiation [42]. These mechanical abnormalities complete Virchow’s triad and interact synergistically with obesity-related hypercoagulability and endothelial dysfunction.

Together, adipose tissue dysfunction, hypercoagulability, endothelial activation, immune-thrombosis, and venous stasis constitute a biologically integrated framework linking obesity to venous thromboembolism (Figure 1). These mechanisms also provide the rationale for exploring whether therapies capable of reversing adipose tissue dysfunction may also reduce thromboembolic risk.

## 4. Weight Loss and Venous Thromboembolic Risk

### 4.1. Effects of Weight Loss on Haemostatic and Inflammatory Pathways

Effective weight reduction consistently improves the haemostatic and inflammatory abnormalities associated with obesity, providing indirect evidence that reducing adipose tissue dysfunction may attenuate thrombotic risk. Reductions in visceral adiposity are accompanied by lower circulating levels of PAI-1, fibrinogen, factor VIII, and markers of thrombin generation, as well as attenuation of chronic low-grade inflammation [33,43]. In addition, weight loss has been associated with a significant reduction in circulating procoagulant extracellular vesicles (formerly referred to as microparticles), particularly those derived from platelets and endothelial cells, suggesting that reversal of adipose tissue dysfunction also mitigates cellular pathways involved in thrombo-inflammatory activation [44,45]. Although these findings strongly support a mechanistic link between weight loss and a less prothrombotic phenotype, biomarkers such as PAI-1, C-reactive protein (CRP), and IL-6 have not been validated as surrogate endpoints for VTE. Consequently, whether these biological improvements translate into fewer clinical thromboembolic events requires evidence based on clinical outcome studies rather than surrogate biomarker changes alone.

### 4.2. Bariatric Surgery as a Clinical Model

Bariatric surgery provides the most informative clinical model for determining whether the biological improvements associated with weight loss translate into clinically meaningful reductions in VTE. However, interpretation of this evidence requires distinguishing the early postoperative period, characterized by transient thrombotic vulnerability, from the long-term effects of sustained weight reduction [46,47,48].

Although bariatric surgery ultimately improves the biological substrate underlying thrombosis, the immediate postoperative period is characterized by substantially increased VTE risk, with thromboembolism remaining a leading cause of postoperative mortality [49]. This apparent paradox illustrates that short-term thrombotic vulnerability and long-term metabolic benefit are not temporally synchronous. Understanding how this transient perioperative risk should be managed is therefore essential when interpreting the long-term thrombotic effects of bariatric surgery. Evidence supporting current thromboprophylaxis strategies is less robust than commonly perceived. A 2022 Cochrane review including seven randomized trials found little or no difference between higher- and standard-dose heparin, between heparin and pentasaccharides, or according to the timing of prophylaxis, with all comparisons supported by low- or very low-certainty evidence [28]. Only combined mechanical and pharmacological prophylaxis demonstrated a potential benefit, based on a single small trial [50]. More recently, dedicated studies have begun to address this evidence gap. A phase II randomized trial showed that postoperative rivaroxaban prophylaxis was associated with a very low incidence of thromboembolic events and an acceptable safety profile after bariatric surgery [51]. Similarly, a prospective cohort from Kyiv reported no clinical or imaging-confirmed VTE events following a step-down strategy from low molecular weight heparin (LMWH) to rivaroxaban, although the absence of a comparator limits causal inference [52].

Once the immediate postoperative period has passed, attention shifts from perioperative thromboprophylaxis to the long-term effects of sustained weight loss. In a population-based matched cohort including more than 13,000 surgical patients followed for a median of 8.4 years, bariatric surgery was associated with a 21% reduction in long-term VTE risk (HR 0.79, 95% CI 0.66–0.96) after exclusion of the immediate postoperative period, representing one of the few studies demonstrating a reduction in adjudicated clinical thromboembolic events rather than improvements in surrogate biomarkers [53]. Importantly, the effect was not uniform. In fact, risk reduction was more pronounced in men (HR 0.59) and in patients with BMI below 50 kg/m^2^, with no detectable benefit in patients with BMI ≥ 50 kg/m^2^ or in those who underwent sleeve gastrectomy specifically. Taken together, these findings provide the strongest clinical support currently available for the concept that sustained weight loss may translate into meaningful reductions in VTE incidence. The similarity between its effect size (HR 0.79) and the active-comparator estimates reported for GLP-1 receptor agonists and tirzepatide (HR 0.78–0.90) is intriguing and supports, although does not prove, the hypothesis that weight loss itself may be a major determinant of thrombotic risk reduction [54]. Whether comparable reductions in VTE risk can also be achieved without surgery is therefore the next logical question.

### 4.3. Pharmacological Weight Loss: Extending the Paradigm Beyond Bariatric Surgery

The demonstration that sustained weight loss following bariatric surgery may reduce long-term VTE risk naturally raises the question of whether comparable benefits can also be achieved through pharmacological weight-loss interventions. Modern incretin-based therapies have fundamentally changed obesity treatment by producing weight reductions previously attainable almost exclusively through bariatric surgery [55].

Semaglutide 2.4 mg once weekly produced mean weight reductions of approximately 15% in the STEP 1 trial [13], while tirzepatide, a dual GIP/GLP-1RA, achieved up to 22.5% in the SURMOUNT-1 trial [14]. Beyond weight loss, these agents improve glycemic control, blood pressure, lipid profiles, hepatic steatosis, and circulating inflammatory markers, supporting their role as comprehensive cardiometabolic therapies rather than purely anti-obesity drugs [56]. These metabolic benefits have translated into meaningful reductions in MACEs, as demonstrated in LEADER [16], SUSTAIN-6 [17], REWIND [18], and SELECT [19], the latter extending cardiovascular benefit to people with obesity without diabetes [19]. Collectively, these findings generated the hypothesis that the favourable vascular effects of incretin-based therapies might also extend to venous thromboembolism [33]. Whether this assumption is justified, however, requires careful evaluation, as the biological mechanisms underlying arterial and venous thrombosis overlap only partially.

Other approved anti-obesity medications, including naltrexone/bupropion, phentermine/topiramate, orlistat, and setmelanotide for monogenic obesity syndromes [57], have not been specifically evaluated for VTE outcomes and lack the extensive cardiometabolic outcome data available for incretin-based therapies. Consequently, the discussion that follows focuses primarily on GLP-1RAs and tirzepatide, while sodium-glucose cotransporter-2 inhibitors (SGLT2-is) are considered mainly as active comparators because they have shown broadly similar observational VTE signals despite producing substantially smaller weight loss. These considerations provide the biological and clinical rationale for examining whether incretin-based therapies exert effects on venous thrombosis beyond those attributable to weight loss alone.

### 4.4. Why Arterial Benefit Does Not Necessarily Predict Venous Benefit

The inference that major adverse cardiovascular event (MACE) reduction with GLP-1RAs necessarily implies protection against VTE cannot be taken for granted. Arterial and venous thrombosis share inflammatory pathways but differ substantially in their dominant biological mechanisms. Arterial thrombosis develops predominantly at sites of atherosclerotic plaque disruption under high shear conditions and is largely platelet-driven, whereas venous thrombosis results primarily from coagulation activation and venous stasis under low-shear conditions [58,59]. Consequently, antiplatelet therapies are central to arterial prevention, whereas anticoagulants remain the cornerstone of VTE prevention and treatment. The cardiovascular outcome trials establishing GLP-1RA benefits enrolled populations at high atherosclerotic risk, predominantly individuals with established coronary, cerebrovascular, or peripheral arterial disease [60]. The clinical profile of patients at elevated VTE risk differs; they more often have thrombophilia, cancer, immobilization, pregnancy, or prior thrombotic events. Risk factor overlap is genuine but partial, and the mechanisms by which GLP-1RAs reduce MACEs, including effects on plaque stability and atherosclerotic inflammation, are not directly applicable to the venous stasis-coagulation axis that drives DVT. Consequently, cardiovascular outcome trials (CVOTs) were neither designed nor adequately powered to evaluate venous thromboembolic events, and any apparent reduction in VTE should therefore be interpreted as exploratory rather than confirmatory.

Nevertheless, these biological differences do not exclude a potential protective effect on venous thrombosis. Immuno-thrombosis, NET formation, tissue factor pathway activation, and inflammatory coagulation represent shared mechanisms through which incretin-based therapies could plausibly influence both arterial and venous disease [61,62]. Whether this biological plausibility translates into clinically meaningful reductions in VTE can only be answered by direct clinical evidence, which is critically appraised in the following sections [2,63]. The available evidence must therefore be interpreted on its own merits rather than inferred from cardiovascular outcome trials.

## 5. Clinical Evidence: Do Incretin-Based Therapies Reduce Venous Thromboembolism?

### 5.1. Mechanistic Evidence Beyond Weight Loss

Experimental studies consistently suggest that GLP-1 receptor activation favourably modulates several pathways implicated in thrombosis. GLP-1-related therapies reduce circulating PAI-1, CRP, and IL-6 levels, improve endothelial nitric oxide bioavailability, attenuate oxidative stress, and reduce platelet activation, thereby providing a biologically coherent rationale for a potential reduction in thrombotic risk [64,65,66]. The anti-aggregatory effect of exenatide was demonstrated by Cameron-Vendrig et al., who showed that GLP-1 receptor activation inhibits platelet aggregation through an endothelial nitric oxide synthase (eNOS)-dependent mechanism under both normoglycemic and hyperglycemic conditions [67]. Similarly, Barale et al. demonstrated that native GLP-1, its degradation product GLP-1 [68], and liraglutide reduced collagen- and arachidonic acid-induced platelet aggregation via enhanced NO/cGMP/PKG signaling together with inhibition of the PI3-K/Akt and MAPK/ERK pathways [68]. Collectively, these findings support the concept that incretin-related peptides can directly influence platelet biology under controlled experimental conditions. However, whether these experimental observations remain clinically relevant once the effect of weight loss itself is taken into account is far less certain. The only randomized study specifically designed to address this question was conducted by Simeone et al., who compared liraglutide with intensive lifestyle intervention in obese individuals with prediabetes or early type 2 diabetes, with both groups achieving the same predefined weight-loss target (−7% of initial body weight) [69]. Thromboxane-dependent platelet activation and lipid peroxidation decreased substantially in both groups, with no significant between-group differences (urinary 11-dehydro-thromboxane (TX)B_2_, *p* = 0.679; 8-iso-PGF_2α_, *p* = 0.985), paralleling comparable improvements in inflammation and glycaemic control. Therefore, when weight loss was held constant, liraglutide conferred no additional antiplatelet benefit beyond lifestyle intervention. This observation substantially changes the interpretation of the experimental literature, suggesting that much of the improvement in platelet function may be attributable to weight loss itself rather than to a drug-specific pharmacological effect. At first glance, these findings appear inconsistent with earlier mechanistic studies. However, the ELAID pilot trial provides a possible explanation [70]. In this placebo-controlled study, liraglutide attenuated platelet aggregation during the first week of treatment, before substantial weight loss had occurred, but this effect was transient and had disappeared during longer follow-up. Taken together with the findings of Simeone et al. [69], these data suggest that weight reduction itself may account for a substantial proportion of the observed improvements in thrombo-inflammatory and platelet-related biomarkers. Although an early, weight-independent effect of GLP-1RAs on platelet function has been suggested, the available evidence is insufficient to establish a clinically relevant direct antithrombotic effect. Accordingly, sustained weight loss currently represents the most plausible mediator of any potential reduction in VTE risk associated with incretin-based therapies. Any additional weight-independent pharmacological contribution remains biologically plausible but unproven and requires further investigation.

Further complexity emerges from the study by Barale et al., which showed that the antiplatelet and antioxidant effects of GLP-1-related peptides were largely absent in subjects with primary hypercholesterolemia and were not restored after four weeks of simvastatin therapy despite normalization of lipid levels [71]. Given that hypercholesterolemia is highly prevalent among individuals with obesity and type 2 diabetes, these findings raise questions regarding the generalizability of experimental platelet studies to the populations most commonly treated with GLP-1RAs. Similarly, the observation that platelet inhibition persisted despite pharmacological blockade of the GLP-1 receptor suggests that at least part of the reported antiplatelet effect may occur through receptor-independent mechanisms, the clinical relevance of which remains uncertain [68]. Together, these observations illustrate the gap that may exist between mechanistic studies performed under controlled experimental conditions and the biological complexity encountered in clinical practice. Overall, the available evidence strongly supports the biological plausibility of an antithrombotic effect of incretin-based therapies but does not yet demonstrate a clinically meaningful drug-specific benefit independent of weight loss. Improvements in PAI-1, CRP, IL-6, and platelet function remain mechanistically informative but are not validated surrogate endpoints for VTE, and previous experience with cardiovascular pharmacology has repeatedly shown that favourable changes in surrogate biomarkers do not necessarily translate into reductions in clinical events [44]. Consequently, mechanistic evidence should currently be regarded as hypothesis-generating rather than confirmatory, underscoring the need for prospective clinical studies evaluating adjudicated VTE outcomes.

### 5.2. Evidence from Clinical Studies

No randomized controlled trial (RCT) has been specifically designed to evaluate VTE as a primary or key secondary endpoint of incretin-based therapy. Consequently, the available clinical evidence must be interpreted according to the strengths and limitations of different study designs rather than as a single body of evidence. In the major CVOTs, including LEADER [16], SUSTAIN-6 [17], REWIND [18], and SELECT [19], VTE events were infrequent, inconsistently reported, and not prospectively adjudicated as dedicated study outcomes. These trials therefore provide robust evidence for cardiovascular benefit but cannot establish whether GLP-1RAs reduce venous thromboembolic events [16,17,18,19]. The randomized evidence regarding VTE, including the apparent discrepancy between randomized and observational findings, is discussed in greater detail below.

#### 5.2.1. Randomized Evidence

A meta-analysis of 21 SUSTAIN and PIONEER randomized trials raised a potential DVT safety signal with semaglutide (RR 3.66, 95% CI 1.09–12.25). However, DVT was assessed as a serious adverse event rather than a prespecified thromboembolic endpoint, events were rare, and the estimate was characterized by a wide confidence interval [72].

More recent meta-analyses of RCTs have not demonstrated a statistically significant reduction in overall VTE with GLP-1RAs, although estimates for individual VTE components have been inconsistent across analyses [73,74].

In a meta-analysis of 27 placebo-controlled trials including 84,003 participants, GLP-1RAs were associated with a numerically lower risk of overall VTE, although the difference did not reach statistical significance (RR 0.70, 95% CI 0.46–1.07). A significant reduction in pulmonary embolism was observed (RR 0.60, 95% CI 0.39–0.94), whereas no significant reduction in DVT was detected (RR 1.21, 95% CI 0.69–2.12) [74].

Conversely, another meta-analysis including 39 randomized trials and 70,499 participants reported no significant effect on overall VTE (OR 1.19, 95% CI 0.94–1.50), no significant association with pulmonary embolism (PE), and a significantly higher incidence of DVT among GLP-1RA-treated participants (OR 1.64, 95% CI 1.14–2.36) [73]. Notably, the DVT signal was more pronounced in trials with treatment duration > 1.5 years (OR 2.32, 95% CI 1.49–3.60) and in CVOTs (OR 2.18, 95% CI 1.36–3.49) [73]. These apparently conflicting findings underscore the uncertainty surrounding VTE estimates derived from currently available randomized trials.

#### 5.2.2. Observational Evidence

In contrast to the inconclusive randomized evidence, an expanding body of contemporary observational research has reported associations between incretin-based therapies and lower rates of venous thromboembolic events across diverse clinical populations. In a large target-trial emulation involving 540,258 propensity-score-matched patients with type 2 diabetes, Chiang et al. reported a lower risk of overall VTE with GLP-1RA therapy compared with dipeptidyl peptidase-4 inhibitors (DPP-4i) (HR 0.78, 95% CI 0.73–0.83), with concordant associations for PE (HR 0.74, 95% CI 0.68–0.82) and DVT (HR 0.81, 95% CI 0.75–0.88) [75]. Similar associations have subsequently been reported in other high-risk populations, including patients with obesity and chronic venous insufficiency, obesity and autoimmune disease, active cancer, and selected perioperative cohorts [76].

Notably, Dai et al., using a target-trial emulation in patients with obesity and autoimmune disease, reported lower rates of VTE (HR 0.83, 95% CI 0.72–0.95) and PE (HR 0.69, 95% CI 0.56–0.86) among GLP-1RA initiators [76]. Micalo et al. similarly observed lower DVT and PE rates at both 1 and 3 years among patients with obesity and chronic venous insufficiency receiving GLP-1RAs [77]. In patients with active cancer, Mahadevan et al. reported a lower incidence of PE compared with metformin (RR 0.61, 95% CI 0.44–0.84) [78]. Additional perioperative studies have reported lower or numerically lower rates of DVT or PE among GLP-1RA users undergoing lumbar laminectomy or total knee arthroplasty [79,80]. Zhang et al. reported significantly lower 90-day rates of DVT, together with fewer postoperative complications and lower mortality, among propensity score-matched GLP-1RA users undergoing diabetic foot reconstruction compared with non-users [81]. Similarly, Heo et al. observed a lower incidence of the composite DVT/PE outcome within 90 days after anterior cervical discectomy and fusion, although this difference disappeared during one-year follow-up [82]. A further propensity score-matched study evaluating common hand procedures also found lower postoperative rates of both DVT and pulmonary embolism among GLP-1RA users [83].

These findings extend the observational signal across populations characterized by substantially different baseline thrombotic risks.

Evidence concerning tirzepatide further illustrates both the potential signal and the importance of comparator selection. In a large new-user cohort of patients with obesity, tirzepatide was associated with a modestly lower risk of VTE than GLP-1RAs (HR 0.90, 95% CI 0.83–0.98), driven predominantly by PE, whereas no significant difference in DVT was observed [84]. Kahkedjian et al. reported substantially lower PE and DVT rates with tirzepatide compared with lifestyle intervention; however, these associations were markedly attenuated when semaglutide was used as an active comparator [85]. This attenuation emphasizes that effect estimates derived from comparisons with non-pharmacological or untreated controls may partly reflect differences in weight loss, metabolic improvement, healthcare engagement, or residual confounding rather than a specific antithrombotic pharmacological effect.

#### 5.2.3. Reconciling Randomized and Observational Evidence

Several factors may explain the inconsistency between randomized and observational data. First, VTE events were uncommon in the RCTs; in the meta-analysis by Chiang et al., the median incidence of VTE was only 1.1 and 2.5 events per 1000 patient-years in the GLP-1RA and placebo groups, respectively [74]. Consequently, even large CVOTs may provide limited statistical power for individual thromboembolic endpoints. Second, these trials were designed primarily to evaluate glycemic, weight-loss, or cardiovascular outcomes rather than VTE, which was generally not a primary efficacy endpoint. Differences in outcome definitions, reporting of DVT and PE, trial populations, comparators, treatment duration, and statistical approaches to rare and zero-event outcomes may therefore substantially influence pooled estimates [73,74].

A further consideration is the temporal relationship between weight loss and thrombotic risk. If a substantial component of any VTE benefit is mediated indirectly through sustained weight reduction and progressive reversal of obesity-related inflammation, hypercoagulability, endothelial dysfunction, and venous stasis, such an effect may emerge gradually. It may be difficult to characterize in trials not specifically designed to assess VTE. Notably, longer treatment duration cannot explain the discrepancy by itself, since one randomized meta-analysis reported a stronger DVT signal in trials lasting >1.5 years [73].

Moreover, the growing body of observational evidence suggesting lower VTE rates among GLP-1RA users must be interpreted in light of the methodological limitations inherent to non-randomized studies. Although these analyses provide valuable real-world information, treatment allocation is not random, and residual confounding remains an important concern even after extensive statistical adjustment [86].

One of the principal sources of bias is the healthy-user effect. Patients prescribed GLP-1RAs often differ systematically from untreated individuals in ways that are difficult to measure completely. They are more likely to receive comprehensive cardiometabolic care, adhere to prescribed therapies, participate in lifestyle interventions, and maintain regular follow-up, all of which may independently reduce thrombotic risk. Consequently, comparisons between GLP-1RA users and untreated or placebo-referent populations may overestimate any true treatment effect. For this reason, active-comparator designs, in which GLP1-RAs are compared with alternative glucose-lowering therapies prescribed to clinically similar patients, represent a substantial methodological advance. As discussed in the previous section, the meta-analysis by Boccatonda et al. [54] largely relied on this strategy by comparing GLP1-RAs with DPP-4is, thereby reducing confounding by indication and healthcare engagement. Nevertheless, even active-comparator studies cannot eliminate residual confounding, since prescribing patterns, disease severity, socioeconomic factors, and physician preferences may still differ between treatment groups. A separate methodological issue is immortal time bias, which occurs when periods during which patients cannot yet experience the study outcome are incorrectly attributed to the treatment group, artificially inflating the apparent protective effect of therapy [87]. Awareness of this bias has substantially improved the methodological quality of contemporary pharmacoepidemiological studies. In particular, target trial emulation, adopted by several of the studies, attempts to reproduce the design principles of a hypothetical randomized trial and is currently considered one of the most robust observational approaches for comparative effectiveness research [88]. Overall, the current observational literature should therefore be viewed hierarchically. Studies comparing GLP-1RAs with untreated populations provide useful exploratory information but remain highly susceptible to confounding. Active-comparator studies employing rigorous causal inference methods currently represent the most reliable observational evidence available, although confirmation from prospective randomized trials with adjudicated VTE outcomes remains essential.

Therefore, randomized and observational findings should be considered complementary rather than selectively prioritized: randomized evidence currently remains inconclusive for overall VTE, whereas observational studies provide a consistent but non-causal signal of potential benefit. Dedicated randomized trials with prespecified and adjudicated VTE endpoints are required to resolve this discrepancy.

#### 5.2.4. Pulmonary Embolism: Differential Clinical Signals and Preliminary Evidence in Established Disease

The distinction between PE and DVT deserves specific consideration when interpreting the available clinical evidence. In the large active-comparator cohort study by Wu et al., including 701,374 propensity-score-matched patients with obesity, tirzepatide was associated with a modestly lower 1-year risk of overall VTE compared with GLP-1RAs (HR, 0.90; 95% CI, 0.83–0.98). This association appeared to be driven predominantly by a lower risk of PE (HR, 0.88; 95% CI, 0.79–0.98), whereas no significant difference was observed for DVT (HR, 0.96; 95% CI, 0.86–1.07) [84]. The biological basis for this apparent PE–DVT discrepancy remains uncertain. Although PE and DVT represent closely related manifestations of the VTE spectrum, differences between these outcomes have also emerged across randomized evidence. One recent meta-analysis of 27 randomized trials reported a lower risk of PE with GLP-1RAs (RR, 0.60; 95% CI, 0.39–0.94), without a significant reduction in DVT (RR, 1.21; 95% CI, 0.69–2.12), whereas another meta-analysis of 39 randomized trials reported an increased risk of DVT (OR, 1.64; 95% CI, 1.14–2.36) without a significant association with PE [73,74]. These inconsistencies may reflect differences in study populations, event definitions and ascertainment, follow-up duration, statistical power, residual confounding in observational studies, or chance rather than a true differential biological effect on pulmonary versus deep venous thrombosis. Accordingly, PE-specific findings should currently be regarded as hypothesis-generating and require confirmation in adequately powered studies with prespecified and independently adjudicated PE and DVT outcomes.

Beyond the potential prevention of incident VTE, preliminary prospective evidence has also explored incretin-based therapy during recovery from established PE. In a small, non-randomized, controlled proof-of-concept study of 22 patients with acute intermediate–high-risk PE, semaglutide (0.25 mg weekly) added to standard-of-care anticoagulation for four weeks was associated with changes in the plasma proteome and improvement in exploratory radiological markers of right ventricular dysfunction [89]. Thrombus burden decreased in both treatment groups, however, and the study was not designed or powered to demonstrate an effect on thrombus resolution or clinical outcomes. These findings should therefore be interpreted as preliminary mechanistic evidence supporting further investigation of incretin-based therapy during PE recovery, rather than as evidence of therapeutic efficacy in acute PE.

### 5.3. Overall Appraisal of the Evidence

Taken together, the available evidence suggests that incretin-based therapies may influence VTE risk, although the direction and magnitude of this effect remain uncertain and the strength of evidence varies substantially across evidence domains [54,73,74,90]. Overall, the convergence of mechanistic, biomarker, and observational evidence provides a coherent rationale for a potential reduction in venous thromboembolic risk associated with incretin-based therapies. However, sustained weight loss and the accompanying reversal of obesity-related thrombotic mechanisms currently represent the most plausible mediators of any potential long-term benefit, whereas a clinically relevant weight-independent antithrombotic effect of incretin receptor activation remains unproven.

These findings should nevertheless be interpreted cautiously, as residual confounding cannot be completely excluded. In addition, part of the evidence discussed in this review, including a recent active-comparator meta-analysis, originates from studies conducted by members of the present author group. Independent external validation is therefore particularly important, and these findings should be confirmed by research groups not involved in the original studies, across different populations and healthcare systems.

Dedicated RCTs with prespecified and adjudicated VTE outcomes, ideally incorporating analyses capable of disentangling weight-loss-mediated from weight-independent effects, are required to establish whether incretin-based therapies truly reduce venous thromboembolic risk (Table 1 and Table 2).

## 6. Clinical Implications

### 6.1. Obesity as a Modifiable VTE Risk Factor

Obesity is one of the few major risk factors for VTE that is potentially reversible. This distinguishes it from many inherited or non-modifiable thrombotic risk factors and raises the clinically relevant question of whether effective obesity treatment should be considered part of a broader strategy for VTE risk reduction. Current VTE risk assessment models (Padua score for medical inpatients and the Caprini score for surgical patients) incorporate BMI as a static variable but do not account for dynamic changes in body weight, adipose tissue dysfunction, or improvements achieved through modern anti-obesity therapies [93].

The evidence reviewed in the preceding sections suggests that sustained weight loss may modify the biological substrate underlying venous thrombosis by attenuating inflammation, hypercoagulability, endothelial dysfunction, and impaired fibrinolysis. However, current evidence remains insufficient to support obesity treatment as a dedicated strategy for VTE prevention. At present, the primary indication for incretin-based therapies remains obesity and its cardiometabolic complications, while any reduction in thromboembolic risk should be regarded as a potential additional benefit rather than an established therapeutic objective.

### 6.2. Patients with Prior VTE: An Emerging Clinical Question

Patients with a history of VTE represent one of the most clinically relevant populations in whom the potential antithrombotic effects of incretin-based therapies warrant further investigation. Obesity is an established predictor of VTE recurrence after anticoagulation withdrawal [25,26], suggesting that persistent adipose tissue dysfunction contributes not only to the first thrombotic event but also to long-term thrombotic risk. The mechanistic and clinical evidence reviewed in the preceding sections raises the possibility that effective and sustained weight reduction could favourably modify this residual risk [94]. Recent active-comparator pharmacoepidemiological studies further support this hypothesis by demonstrating lower VTE rates among patients receiving GLP-1RAs compared with DPP-4is [54]. Similarly, the limited literature on patients with previous VTE undergoing bariatric surgery focuses primarily on perioperative anticoagulation management rather than on the long-term effect of weight reduction on recurrent thrombosis [95]. Consequently, no evidence currently supports modifying decisions regarding the duration or intensity of anticoagulation based on successful weight loss alone.

At present, patients with prior VTE should receive incretin-based therapies according to established metabolic indications, including obesity and type 2 diabetes, while any potential reduction in recurrent thromboembolic risk should be regarded as an additional, unconfirmed benefit. Dedicated prospective studies in patients with previous VTE should be considered a research priority, as this population is likely to derive the greatest absolute benefit if the protective association suggested by current evidence is ultimately confirmed.

### 6.3. Challenges of Anticoagulant Therapy in Obesity and Extreme Body Weight

Obesity and extreme body weight also raise specific considerations for the prevention and treatment of VTE (Table 3). Alterations in volume of distribution, renal clearance, and drug exposure may affect the pharmacokinetic and pharmacodynamic behavior of anticoagulant agents, while patients with severe obesity have historically been underrepresented in pivotal randomized trials. These concerns initially led to caution regarding fixed-dose direct oral anticoagulants (DOACs) in patients with body weight > 120 kg or BMI > 40 kg/m^2^. However, accumulating evidence has progressively reduced this uncertainty. Updated guidance from the International Society on Thrombosis and Haemostasis supports the use of standard-dose apixaban and rivaroxaban for VTE treatment and prevention in patients with obesity, including those with BMI > 40 kg/m^2^ or weight > 120 kg, whereas evidence remains less robust for dabigatran and edoxaban [96].

Recent observational evidence and meta-analyses are broadly reassuring. In patients with morbid obesity and VTE, DOACs appear at least comparable with vitamin K antagonists (VKAs) for prevention of recurrent VTE and may be associated with lower bleeding rates. A recent meta-analysis including more than 30,000 patients with morbid obesity found lower rates of recurrent VTE and major bleeding with DOACs compared with warfarin, with similar favourable findings for apixaban and rivaroxaban. Even more recent pooled data involving over 44,000 patients similarly suggest that standard-dose DOACs are effective and safe in this population, although the evidence remains predominantly observational [97,98].

Parenteral anticoagulation presents different challenges. Therapeutic LMWH is generally dosed according to actual body weight, but extreme obesity can lead to high absolute doses and uncertainty regarding whether laboratory monitoring is useful. Although anti-factor Xa monitoring has historically been considered in selected patients at extreme body weight, contemporary systematic reviews have found weak correlations between anti-Xa levels and clinical outcomes and limited evidence that routine anti-Xa-guided dose adjustment improves safety or efficacy. Accordingly, routine monitoring is not supported, although selective testing may be considered when altered drug elimination, unexpected clinical response, or other major pharmacokinetic concerns are present [99,100].

These issues are clinically relevant to the present discussion because substantial weight loss induced by incretin-based therapies may change body weight, renal function, comorbidity burden, and potentially anticoagulant exposure over time. However, there is currently insufficient evidence to determine whether pharmacologically induced weight loss requires systematic modification of anticoagulant dosing or alters the long-term balance between recurrent VTE and bleeding. Importantly, any potential reduction in thromboembolic risk associated with incretin-based therapy should be considered complementary to, and not a substitute for, guideline-directed anticoagulant prophylaxis or treatment when clinically indicated.

### 6.4. Practical Considerations

Gastrointestinal adverse effects during initiation of incretin-based therapies may lead to dehydration in patients who are already predisposed to thrombosis. Although simple, maintaining adequate hydration represents an important practical measure that should not be overlooked. In patients with sarcopenic obesity, preservation of lean mass through adequate protein intake and resistance exercise may be particularly relevant, as excessive muscle loss could theoretically impair calf-muscle pump function and partially offset the hemodynamic benefits of weight reduction. This recommendation is also consistent with broader evidence supporting the safety and potential benefits of physical activity after a venous thromboembolic event [101]. As highly effective anti-obesity therapies become more widely used, multidisciplinary collaboration between obesity specialists, internists, and thrombosis physicians will become increasingly important. Particular attention should be paid to patients receiving long-term anticoagulation, in whom substantial pharmacologically induced weight loss raises unresolved questions regarding DOAC dosing, as discussed above. Likewise, the perioperative setting deserves further investigation. Patients with obesity and diabetes undergoing elective surgery represent a population in whom baseline thrombotic risk, surgical trauma, and postoperative immobilization converge, while preliminary observational studies suggest that pre-existing GLP-1 receptor agonist therapy may be associated with a more favourable perioperative thrombotic profile [81,82,83]. However, these findings remain exploratory and should not currently influence perioperative thromboprophylaxis strategies. Overall, the available evidence supports cautious optimism rather than therapeutic enthusiasm. The biological rationale is compelling, mechanistic findings are broadly supportive, and contemporary active-comparator studies provide a clinically relevant observational signal suggesting a potential reduction in venous thromboembolic risk with incretin-based therapies. However, randomized evidence remains inconclusive and partly conflicting, and no randomized trial has specifically evaluated VTE prevention as a primary outcome. Moreover, sustained weight loss currently represents the most plausible mediator of any potential long-term benefit, whereas a clinically relevant direct pharmacological antithrombotic effect remains unproven. Accordingly, incretin-based therapies should continue to be prescribed according to established metabolic indications, and any reduction in thromboembolic risk should presently be regarded as a potential but unconfirmed additional benefit rather than an established therapeutic indication. Practice should not run ahead of confirmatory trial data, but neither should the growing body of convergent evidence be dismissed as merely speculative [102].

### 6.5. Limitations of This Review

This review has several limitations. First, it was designed as a narrative review informed by a structured literature search rather than as a systematic review; therefore, no prospective protocol was registered, no formal systematic risk-of-bias assessment was performed, and study selection may be subject to selection bias. Second, the available clinical evidence is highly heterogeneous in terms of study design, patient populations, therapeutic agents, comparators, follow-up duration, and VTE definitions and ascertainment, limiting direct comparisons across studies. Moreover, VTE was not a prespecified or adjudicated outcome in many RCTs, while much of the contemporary evidence derives from observational studies that remain susceptible to residual confounding, confounding by indication, exposure and outcome misclassification, and potential overlap between real-world data sources. Moreover, the limited number of VTE events in randomized studies and the absence of dedicated VTE prevention trials preclude firm conclusions regarding causality or clinical efficacy. In addition, current evidence does not allow the effects of sustained weight loss to be reliably separated from potential weight-independent pharmacological actions of incretin receptor activation.

## 7. Research Priorities

The most important limitation of the current evidence base is the absence of RCTs specifically designed to evaluate VTE. Although active-comparator pharmacoepidemiological studies have substantially strengthened the available evidence by reducing many of the biases inherent to conventional observational analyses [54], residual confounding cannot be completely excluded. Likewise, cardiovascular outcome trials were neither powered nor designed to assess VTE, and venous thromboembolic events were infrequent, inconsistently reported, and generally not prospectively adjudicated [16,17,18,19]. Consequently, definitive confirmation of a protective effect of incretin-based therapies requires a dedicated RCT with VTE as a prespecified primary endpoint. Such a trial should enroll individuals at sufficiently high thrombotic risk to ensure an adequate event rate and maximize clinical relevance. Populations of particular interest include patients with severe obesity, previous DVT or pulmonary embolism, active cancer, or inherited thrombophilia, rather than the predominantly high-atherosclerotic-risk populations included in existing cardiovascular outcome trials. Prospective adjudication of both DVT and PE should be incorporated, together with prespecified subgroup analyses according to baseline obesity severity, previous VTE, and concomitant anticoagulant therapy. In addition to clinical outcomes, mechanistic substudies should be embedded within future randomized trials. Serial assessment of inflammatory, coagulation, endothelial, and immune-thrombotic biomarkers may help clarify which biological pathways are modified by treatment and whether changes in these markers parallel reductions in thromboembolic events. Although these biomarkers cannot currently be considered validated surrogate endpoints for VTE [44], their integration into adequately powered clinical trials would provide valuable mechanistic insight while avoiding the interpretative limitations of biomarker-only studies.

An equally important research priority is to determine whether any reduction in VTE risk associated with incretin-based therapies is primarily driven by weight loss itself or by direct pharmacological effects on thrombosis-related pathways. Current evidence does not allow these mechanisms to be clearly separated. Notably, the only randomized human study specifically designed to control for the magnitude of weight loss found no additional reduction in platelet activation with liraglutide compared with lifestyle intervention when equivalent weight loss was achieved [69]. Future studies should therefore compare incretin-based therapies with non-incretin interventions achieving similar weight loss, or directly compare active treatments producing equivalent reductions in body weight but differing in incretin receptor activation. Such studies will be essential to determine whether thromboembolic protection reflects reversal of obesity-related pathophysiology, direct pharmacological effects of incretin receptor activation, or a combination of both.

Until dedicated randomized trials become available, high-quality observational studies will continue to play an important role in advancing the field. Future pharmacoepidemiological research should preferentially adopt active-comparator, new-user designs and target trial emulation methodologies, which currently provide the most robust observational evidence for comparative effectiveness [54]. Larger collaborative cohorts should also allow more detailed analyses according to obesity severity, previous VTE, cancer, and other high-risk clinical settings [5]. Several clinically relevant questions also remain unresolved. Whether the apparent differences between pulmonary embolism and deep vein thrombosis observed in some recent analyses reflect true biological differences or chance findings requires further investigation. Likewise, the comparative effects of newer agents, including dual GIP/GLP-1 receptor agonists, should be evaluated in studies directly comparing different incretin-based therapies [84]. Finally, prospective pharmacokinetic studies are needed to determine whether substantial pharmacologically induced weight loss influences DOAC exposure and clinical outcomes in anticoagulated patients.

Although incretin-based therapies have emerged as a promising pharmacological strategy for reducing obesity-related thrombotic risk, they are unlikely to constitute the only effective approach. Future research should also clarify how incretin-based therapies fit within broader strategies for cardiometabolic risk reduction, including sustained weight loss, physical activity, and optimization of metabolic risk factors. Whether these interventions exert complementary or overlapping effects on venous thrombosis remains largely unknown. Ultimately, VTE prevention in obesity will likely require a comprehensive strategy integrating sustained weight reduction, optimization of cardiometabolic risk factors, increased physical activity, and appropriate thromboprophylaxis in selected high-risk individuals. Defining the contribution of each intervention, alone or in combination, represents an important challenge for future clinical research.

Future studies should investigate whether substantial incretin-induced weight loss modifies anticoagulant pharmacokinetics, dosing requirements, drug exposure, or the long-term balance between recurrent VTE and bleeding, particularly in patients transitioning from severe obesity to substantially lower body-weight categories.

## 8. Conclusions

Obesity promotes VTE through the convergence of adipose tissue dysfunction, chronic inflammation, endothelial dysfunction, hypercoagulability, impaired fibrinolysis, immunothrombosis, and venous stasis. Although several of these mechanisms overlap with atherothrombosis, important biological differences between arterial and venous thrombosis preclude direct extrapolation of the established cardiovascular benefits of incretin-based therapies to VTE. Mechanistic and translational studies provide biological plausibility for a potential antithrombotic effect, while contemporary active-comparator pharmacoepidemiological studies suggest lower VTE rates among patients receiving GLP-1RAs compared with DPP-4is. However, meta-analyses of randomized trials remain inconclusive and have yielded partly conflicting findings for overall VTE and its individual components, preventing definitive conclusions regarding clinical efficacy. Current evidence suggests that sustained weight loss is the most plausible mediator of any potential long-term reduction in VTE risk, through the progressive reversal of obesity-related prothrombotic abnormalities. Although weight-independent effects of incretin receptor activation on inflammation, endothelial function, platelet activation, and coagulation pathways remain biologically plausible, a clinically relevant direct pharmacological antithrombotic effect has not been established. Accordingly, incretin-based therapies should continue to be prescribed according to established metabolic indications, and any reduction in VTE risk should currently be regarded as a potential but unconfirmed additional benefit rather than an established therapeutic indication. Dedicated prospective RCTs with prespecified and adjudicated VTE outcomes, clinically relevant comparators, and embedded mechanistic assessments are needed to determine whether these therapies reduce VTE risk, disentangle weight-loss-mediated from weight-independent effects, and identify patient populations in whom any potential benefit may be greatest.

## Figures and Tables

**Figure 1:**
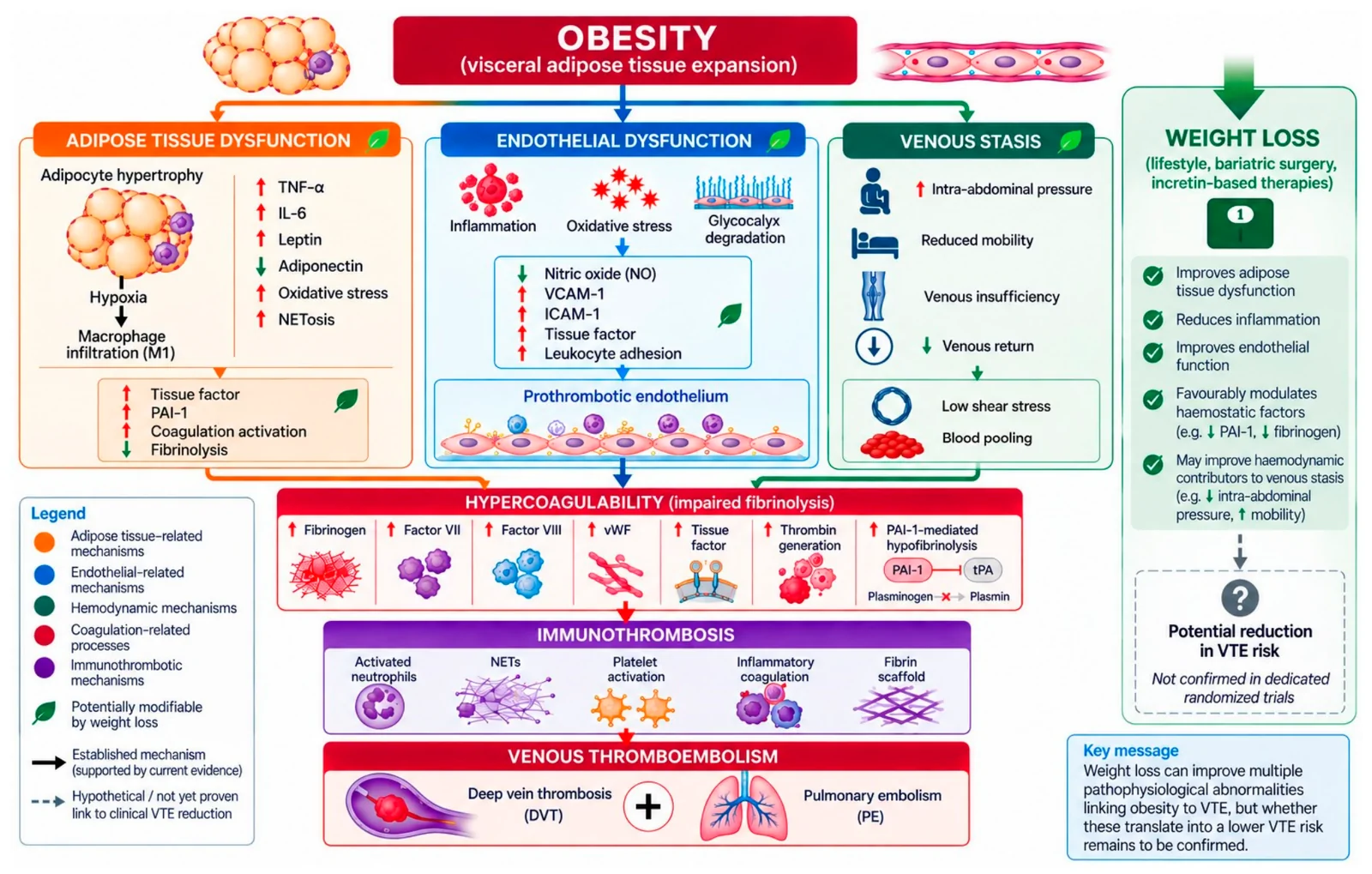
Mechanisms linking obesity to venous thromboembolism and their potential modification by weight loss. Visceral adipose tissue expansion promotes adipose tissue dysfunction, endothelial dysfunction, and venous stasis, which converge to generate hypercoagulability, impaired fibrinolysis, and immunothrombosis, ultimately favouring venous thromboembolism. Adipocyte hypertrophy promotes tissue hypoxia, macrophage infiltration, altered adipokine secretion, and chronic low-grade inflammation. At the same time, endothelial activation is associated with oxidative stress, glycocalyx degradation, reduced nitric oxide bioavailability, increased expression of adhesion molecules and tissue factor, and a prothrombotic endothelial phenotype. Increased intra-abdominal pressure, reduced mobility, and venous insufficiency further contribute to venous stasis and low-shear conditions. Colours identify the major mechanistic domains, whereas green leaf symbols indicate pathways that may be favourably modified by weight loss. Solid arrows represent established or well-supported mechanistic relationships, whereas the dashed arrow denotes the hypothesized translation of weight-loss-induced biological improvements into reduced clinical VTE risk. Although sustained weight loss improves several inflammatory, endothelial, haemostatic, and metabolic abnormalities associated with obesity, whether these changes translate into a reduction in clinical VTE events has not yet been confirmed in dedicated randomized trials. Abbreviations: ↑, increased; ↓, decreased; DVT, deep vein thrombosis; ICAM-1, intercellular adhesion molecule-1; IL-6, interleukin-6; NETs, neutrophil extracellular traps; NO, nitric oxide; PAI-1, plasminogen activator inhibitor-1; PE, pulmonary embolism; TNF-α, tumor necrosis factor alpha; tPA, tissue plasminogen activator; VCAM-1, vascular cell adhesion molecule-1; VTE, venous thromboembolism; vWF, von Willebrand factor.

**Table 1:** Hierarchical appraisal of the current evidence on incretin-based therapies and venous thromboembolic risk.

Evidence Domain	Main Findings	Study Design	Strength of Evidence
Mechanistic studies	Antiplatelet, endothelial, anti-inflammatory and profibrinolytic effects consistently demonstrated	Experimental and ex vivo studies	Low (biological plausibility)
Biomarker studies	Reduction in inflammatory and prothrombotic biomarkers; improved endothelial function	Mechanistic clinical studies, RCT sub-studies	Moderate (no validated VTE surrogate)
RCTs and meta-analyses of RCTs	No significant reduction in overall VTE; conflicting signals for individual components (possible PE reduction vs. possible DVT increase)	Randomized trials/meta-analyses	Moderate, but limited by low event rates and VTE not being a primary endpoint
Active-comparator studies	Lower VTE risk versus DPP-4 inhibitors; generally similar risk versus SGLT2 inhibitors	Active-comparator observational studies	Moderate-High
Pharmacovigilance	No excess thrombotic safety signal	Spontaneous reporting analyses	Low
Conventional observational studies	Generally favourable but heterogeneous findings	Retrospective cohort studies	Low
Dedicated VTE RCTs	No trial specifically designed for VTE prevention	—	Evidence gap

Abbreviations: DPP-4, dipeptidyl peptidase-4; GLP-1, glucagon-like peptide-1; RCT, randomized controlled trial; SGLT2, sodium-glucose cotransporter-2; VTE, venous thromboembolism.

**Table 2:** Summary of original studies and meta-analyses evaluating incretin-based therapies and venous thromboembolic outcomes, ordered chronologically by year of publication. Original studies include comparative observational cohorts, target trial emulations, prospective interventional evidence, and pharmacovigilance analyses, whereas evidence syntheses are reported separately. Differences in populations, comparators, outcome definitions, follow-up duration, and VTE ascertainment should be considered when comparing effect estimates. Observational and pharmacovigilance associations should not be interpreted as proof of causality or preventive efficacy.

**A. Major Studies Evaluating Incretin-Based Therapies and Venous Thromboembolic Outcomes**							
**Study**	**Study Design**	**Population**	**Comparator**	**Follow-Up**	**VTE Outcome**	**Main Findings**	**Major Limitations**
Kim et al., 2025 [79]	Retrospective matched cohort; administrative claims database	Morbid obesity (BMI ≥ 40 kg/m^2^) undergoing primary TKA	Perioperative GLP-1RA use vs. non use	90 days for medical outcomes	PE	PE < 0.4% vs. 0.6%; *p* = 0.050; lower overall medical complications and readmissions with GLP-1RA	PE secondary outcome; very low event rate; borderline significance; claims-based ascertainment; residual confounding; selected perioperative population
Chiang et al., 2025 [75]	Target trial emulation; new-user, propensity-score-matched real-world cohort	T2DM; 540,258 matched patients (270,129/group)	GLP-1RA vs. DPP-4i	12 months	Composite VTE (primary); PE and DVT	VTE HR 0.78 (95% CI 0.73–0.83); PE HR 0.74 (0.68–0.82); DVT HR 0.81 (0.75–0.88)	Observational design; residual confounding; EHR/ICD-based outcomes; no adherence or longitudinal weight-loss data; T2DM population
Mahadevan et al., 2026 [78]	Retrospective real-world cohort; propensity-score matched	T2DM and active cancer receiving systemic anticancer therapy; 3551/group after matching	GLP-1RA vs. metformin	NR	PE	PE RR 0.608 (95% CI 0.440–0.840; *p* = 0.004)	PE secondary outcome; no composite VTE/DVT analysis; residual confounding; performance status and adherence unavailable; selected cancer population
Marzook et al., 2026 [80]	Retrospective propensity-score-matched cohort	Patients undergoing lumbar laminectomy; 4220/group	Preoperative GLP-1RA use vs. nonuse	180 days	DVT	DVT 0.6% vs. 1.2%; RR 0.496 (95% CI 0.308–0.801)	Observational; residual confounding; EHR-based ascertainment; selected perioperative population; DVT only
Micalo et al., 2026 [77]	Retrospective multicenter cohort; TriNetX; 1:1 PSM	Obesity + chronic venous insufficiency; 24,758 matched patients (12,379/group)	GLP-1RA vs. nonuse	1 and 3 years	DVT and PE	DVT: HR 0.54 (0.42–0.71) at 1 y and 0.63 (0.52–0.77) at 3 y; PE: HR 0.43 (0.27–0.66) and 0.69 (0.52–0.90)	Retrospective; residual confounding; ICD-based outcomes; no dose/adherence or longitudinal weight data; possible immortal-time bias; CVI-specific population
Dai et al., 2026 [76]	Target trial emulation, new-user design; OneFlorida+ EHR; time-dependent PSM	Obesity/eligible for anti-obesity pharmacotherapy + autoimmune disease; 26,408 matched patients (13,204/group)	GLP-1RA initiation vs. non-use	Until event/death/loss to follow-up/study end	VTE and PE (primary)	VTE HR 0.83 (0.72–0.95); PE HR 0.69 (0.56–0.86)	Observational; residual confounding; heterogeneous autoimmune diseases; prior VTE permitted; inability to distinguish weight-mediated from direct pharmacological effects
Kahkedjian et al., 2026 [85]	Retrospective population-based cohort; TriNetX; 1:1 PSM	T2DM + overweight/obesity; 235,200 matched patients (117,600/group)	Tirzepatide vs. lifestyle intervention; sensitivity analysis vs. semaglutide	30–365 days	PE, DVT, superficial venous thrombosis	vs lifestyle: PE HR 0.258 (0.222–0.299); DVT HR 0.361 (0.322–0.406); vs. semaglutide: DVT HR 0.828 (0.730–0.939); PE HR 0.857 (0.722–1.017), NS	Residual confounding; EHR-based non-adjudicated outcomes; short follow-up; no granular longitudinal weight, dose or adherence data
Wu et al., 2026 [84]	Active-comparator, new-user retrospective cohort; TriNetX; 1:1 PSM	Adults with obesity; 701,374 matched patients (350,687/group)	Tirzepatide vs. GLP-1RA	1 year	VTE (primary); PE and DVT	VTE HR 0.90 (0.83–0.98); PE HR 0.88 (0.79–0.98); DVT HR 0.96 (0.86–1.07), NS; no clear VTE advantage vs. semaglutide in sensitivity analysis	Residual/temporal confounding; coding/exposure misclassification; adherence and weight changes incompletely captured; GLP-1RAs analyzed as a class
Nanah et al., 2026 [91]	Retrospective multicenter cohort; TriNetX; 1:1 PSM	Obesity + IBD; 3478/group after matching	GLP-1RA vs. bariatric surgery	Mean 648.8 vs. 1346.2 days	DVT and PE	DVT 1.0% vs. 4.2%, OR 0.24 (0.16–0.35); PE 0.8% vs. 2.9%, OR 0.27 (0.17–0.41)	Markedly unequal follow-up; bariatric surgery introduces perioperative VTE risk; residual confounding; ICD-based outcomes; comparison cannot establish weight-independent drug effect
Samaranayake et al., 2026 [89]	Prospective, nonrandomized, controlled, open-label proof-of-concept study	22 patients with acute intermediate–high-risk PE	Semaglutide + anticoagulation (*n* = 8) vs. anticoagulation alone (*n* = 14)	4 weeks	Thrombus burden and RV recovery after established PE	Thrombus burden decreased in both groups; RV/LV and mPA/Ao ratios improved significantly only with semaglutide; exploratory MMP-9/proteomic changes	Very small sample; nonrandomized/open-label; short follow-up; underpowered for clinical outcomes; evaluates recovery after PE rather than incident VTE prevention
Mansory & Radhwi, 2026 [92]	Pharmacovigilance disproportionality analysis; FAERS 2020–2025	1618 unique thrombotic AE reports involving GLP-1RAs	All other FAERS drugs; secondary comparisons with orlistat and SGLT2i	N/A	Arterial, venous and mixed thrombotic AEs	No disproportionate overall thrombotic reporting: ROR 0.67 (0.64–0.71); venous events ROR 0.53 (0.48–0.59); venous events vs. orlistat OR 0.27	Spontaneous reporting; no exposure denominator; reporting/under-reporting bias; confounding by indication; cannot estimate incidence, comparative clinical risk or efficacy
**B. Systematic Reviews and Meta-Analyses**							
**Study**	**Study Design**	**Population/Studies**	**Comparator**	**Follow-Up**	**VTE Outcome**	**Main Findings**	**Major Limitations**
Yin et al., 2021 [72]	Meta-analysis of 21 randomized SUSTAIN and PIONEER trials	12,260 semaglutide users; 14,176 comparators with T2DM	Semaglutide vs. placebo, non-semaglutide GLP-1RA or non-GLP-1RA therapy	Mostly ≤ 1 year	DVT as serious adverse event	Higher DVT risk: RR 3.66 (95% CI 1.09–12.25; I^2^ = 0%; *p* = 0.036)	DVT safety/secondary endpoint; rare events; very wide CI and limited power; no overall VTE or PE estimate; mostly short follow-up
Chiang et al., 2025—Meta-Analysis [74]	Systematic review/meta-analysis of 27 placebo-controlled RCTs	84,003 participants	GLP-1RA vs. placebo	Variable	Overall VTE, PE, DVT	Overall VTE: RR 0.70 (0.46–1.07), NS; PE RR 0.60 (0.39–0.94); no significant DVT reduction	Low absolute event rates; VTE generally not prespecified/adjudicated; heterogeneous populations/follow-up; limited statistical power
Liu et al., 2025 [73]	Systematic review/meta-analysis of 39 RCTs	70,499 participants	GLP-1RA vs. placebo or active comparator	Variable	Overall VTE, PE, DVT	Overall VTE: OR 1.19 (0.94–1.50), NS; PE NS; DVT OR 1.64 (1.14–2.36)	Rare events; VTE not primary endpoint; heterogeneous populations and comparators; estimates affected by trial duration and rare-event methodology
Boccatonda et al., 2026 [54]	Systematic review/meta-analysis of 6 active-comparator studies	New-user cohorts, target-trial emulation, nested case–control and randomized evidence	GLP-1RA/SGLT2i vs. DPP-4i; GLP-1RA vs. SGLT2i	Variable	VTE, PE, DVT	GLP-1RA vs. DPP-4i: HR 0.78 (0.73–0.83); SGLT2i vs. DPP-4i: HR 0.72 (0.62–0.82); GLP-1RA vs. SGLT2i: HR 1.02 (0.84–1.23), NS	Only 6 studies; predominantly observational evidence; heterogeneous designs; residual confounding

**Abbreviations:** AE, adverse event; CI, confidence interval; CVI, chronic venous insufficiency; DPP-4i, dipeptidyl peptidase-4 inhibitor; DVT, deep vein thrombosis; EHR, electronic health record; FAERS, FDA Adverse Event Reporting System; GLP-1RA, glucagon-like peptide-1 receptor agonist; HR, hazard ratio; IBD, inflammatory bowel disease; N/A, not applicable; NR, not reported; NS, not statistically significant; OR, odds ratio; PE, pulmonary embolism; PSM, propensity-score matching; ROR, reporting odds ratio; RR, risk ratio; SGLT2i, sodium-glucose cotransporter-2 inhibitor; T2DM, type 2 diabetes mellitus; TKA, total knee arthroplasty; VTE, venous thromboembolism.

**Table 3:** Practical management of anticoagulant therapy in patients with obesity and venous thromboembolism. Practical recommendations are based primarily on the updated International Society on Thrombosis and Haemostasis (ISTH) guidance and contemporary evidence on anticoagulation in obesity.

Clinical Scenario	Practical Approach	Key Considerations
Obesity (BMI 30–40 kg/m^2^)	DOACs at standard approved doses can generally be used for VTE treatment and secondary prevention	No dose adjustment. DOACs are generally preferred over VKAs when otherwise appropriate.
Severe obesity (BMI > 40 kg/m^2^ or weight > 120 kg)	Standard-dose apixaban or rivaroxaban are appropriate oral anticoagulant options	Current evidence is most robust for rivaroxaban and apixaban. VKA and weight-based LMWH remain alternatives.
Extreme obesity (e.g., weight > 150 kg or BMI ≥ 50 kg/m^2^)	Individualized assessment; standard-dose apixaban/rivaroxaban may still be considered, but evidence becomes increasingly limited at extreme weights	Consider renal function, bleeding risk, interacting drugs, adherence, and clinical course. VKA or LMWH may be reasonable alternatives when uncertainty is substantial.
Routine DOAC drug-level monitoring	Not routinely recommended	No clinically validated therapeutic range is available for DOAC concentrations in VTE. Measurement may be considered in selected circumstances in which absorption or exposure is questioned.
LMWH	Use weight-based dosing according to the specific LMWH regimen/product recommendations	Very high absolute doses may occur in severe obesity. Empirical dose capping should not be routinely applied without a specific clinical rationale.
UFH	Use an IV weight-based dosing nomogram with subsequent dose adjustment according to aPTT or anti-Xa activity	UFH response is highly variable and requires frequent laboratory monitoring. In class ≥ 2 obesity, conventional total-body-weight–based nomograms may result in relative overdosing; evidence for optimal dosing at extreme body weight remains limited.
Fondaparinux	Use body-weight–adjusted dosing for acute VTE: 5 mg once daily if <50 kg, 7.5 mg once daily if 50–100 kg, and 10 mg once daily if >100 kg	The 10-mg once-daily dose is recommended for patients > 100 kg; however, clinical evidence becomes limited at very high/extreme body weights. Fondaparinux is predominantly renally eliminated and should not be used in severe renal impairment (CrCl < 30 mL/min).
Vitamin K antagonists	Remain an effective alternative irrespective of body weight	INR-guided dosing avoids uncertainty related to fixed dosing at extreme body weight, but requires monitoring and is affected by diet, interactions, and treatment burden.
Early period after bariatric surgery	Prefer parenteral anticoagulation during the early postoperative phase when therapeutic anticoagulation is required	Altered gastrointestinal anatomy may impair DOAC absorption. ISTH guidance suggests considering transition to VKA or a DOAC only after the initial parenteral phase; assessment of DOAC absorption may be appropriate if a DOAC is subsequently used.
Major weight loss during long-term anticoagulation	Reassess the patient clinically, but do not routinely change DOAC dose solely because of weight loss	Reassess renal function, body weight, comorbidities, concomitant medications, bleeding risk, and treatment indication. Evidence does not currently support systematic DOAC dose modification after pharmacologically induced weight loss.

Abbreviations: BMI, body mass index; DOAC, direct oral anticoagulant; INR, international normalized ratio; LMWH, low-molecular-weight heparin; UFH, unfractionated heparin; VKA, vitamin K antagonist; VTE, venous thromboembolism.

## Data Availability

No new data were created or analyzed in this study. Data sharing is not applicable to this article.

## References

[1] (2024). Worldwide trends in underweight and obesity from 1990 to 2022: A pooled analysis of 3663 population-representative studies with 222 million children, adolescents, and adults. Lancet.

[2] Piazza G., Goldhaber S.Z. (2010). Venous thromboembolism and atherothrombosis: An integrated approach. Circulation.

[3] Zhen K., Tao Y., Xia L., Wang S., Gao Q., Wang D., Chen Z., Meng X., Kang Y., Fan G. (2025). Epidemiology of pulmonary embolism in China, 2021: A nationwide hospital-based study. Lancet Reg. Health West. Pac..

[4] Hotoleanu C. (2020). Association between obesity and venous thromboembolism. Med. Pharm. Rep..

[5] Boccatonda A., Cicero A.F.G. (2026). Multiparametric and multi-organ ultrasound assessment of adiposity for cardiovascular risk stratification. Eur. J. Intern. Med..

[6] Rosendaal F.R. (2005). Venous thrombosis: The role of genes, environment, and behavior. Hematol. Am. Soc. Hematol. Educ. Program..

[7] Boccatonda A., Bray A., Marozzi M.S., Cipollone A., Lauletta G., D’Ardes D., Cicco S., Cipollone F. (2026). From liver to cardiovascular system: The MASLD-ASCVD continuum. Intern. Emerg. Med..

[8] Healy B., Levin E., Perrin K., Weatherall M., Beasley R. (2010). Prolonged work- and computer-related seated immobility and risk of venous thromboembolism. J. R. Soc. Med..

[9] Braithwaite I., Healy B., Cameron L., Weatherall M., Beasley R. (2016). Venous thromboembolism risk associated with protracted work- and computer-related seated immobility: A case-control study. JRSM Open.

[10] Johannesen C.D.L., Flachs E.M., Ebbehøj N.E., Marott J.L., Jensen G.B., Nordestgaard B.G., Schnohr P., Bonde J.P.E. (2020). Sedentary work and risk of venous thromboembolism. Scand. J. Work. Environ. Health.

[11] Baccarelli A., Martinelli I., Zanobetti A., Grillo P., Hou L.F., Bertazzi P.A., Mannucci P.M., Schwartz J. (2008). Exposure to particulate air pollution and risk of deep vein thrombosis. Arch. Intern. Med..

[12] Di Blasi C., Renzi M., Michelozzi P., De’ Donato F., Scortichini M., Davoli M., Forastiere F., Mannucci P.M., Stafoggia M. (2022). Association between air temperature, air pollution and hospital admissions for pulmonary embolism and venous thrombosis in Italy. Eur. J. Intern. Med..

[13] Wilding J.P.H., Batterham R.L., Calanna S., Davies M., Van Gaal L.F., Lingvay I., McGowan B.M., Rosenstock J., Tran M.T.D., Wadden T.A. (2021). Once-Weekly Semaglutide in Adults with Overweight or Obesity. N. Engl. J. Med..

[14] Jastreboff A.M., Aronne L.J., Ahmad N.N., Wharton S., Connery L., Alves B., Kiyosue A., Zhang S., Liu B., Bunck M.C. (2022). Tirzepatide Once Weekly for the Treatment of Obesity. N. Engl. J. Med..

[15] Boccatonda A., D’Ardes D., Brighenti A., Cipollone A., Simeone P.G., Guagnano M.T., Serra C., Santilli F., Cipollone F. (2026). GLP-1-based therapies and limb outcomes in PAD: A systematic review and meta-analysis of real-world studies. Diabetes Res. Clin. Pract..

[16] Marso S.P., Daniels G.H., Brown-Frandsen K., Kristensen P., Mann J.F., Nauck M.A., Nissen S.E., Pocock S., Poulter N.R., Ravn L.S. (2016). Liraglutide and Cardiovascular Outcomes in Type 2 Diabetes. N. Engl. J. Med..

[17] Marso S.P., Bain S.C., Consoli A., Eliaschewitz F.G., Jódar E., Leiter L.A., Lingvay I., Rosenstock J., Seufert J., Warren M.L. (2016). Semaglutide and Cardiovascular Outcomes in Patients with Type 2 Diabetes. N. Engl. J. Med..

[18] Gerstein H.C., Colhoun H.M., Dagenais G.R., Diaz R., Lakshmanan M., Pais P., Probstfield J., Riesmeyer J.S., Riddle M.C., Rydén L. (2019). Dulaglutide and cardiovascular outcomes in type 2 diabetes (REWIND): A double-blind, randomised placebo-controlled trial. Lancet.

[19] Lincoff A.M., Brown-Frandsen K., Colhoun H.M., Deanfield J., Emerson S.S., Esbjerg S., Hardt-Lindberg S., Hovingh G.K., Kahn S.E., Kushner R.F. (2023). Semaglutide and Cardiovascular Outcomes in Obesity without Diabetes. N. Engl. J. Med..

[20] Campello E., Boccatonda A., Napolitano A., Simion C., Simioni P. (2026). The arterial-venous continuum: Bridging atherosclerosis and chronic venous disease through endothelial dysfunction. Intern. Emerg. Med..

[21] Holst A.G., Jensen G., Prescott E. (2010). Risk factors for venous thromboembolism: Results from the Copenhagen City Heart Study. Circulation.

[22] Pomp E.R., le Cessie S., Rosendaal F.R., Doggen C.J. (2007). Risk of venous thrombosis: Obesity and its joint effect with oral contraceptive use and prothrombotic mutations. Br. J. Haematol..

[23] Rahmani J., Haghighian Roudsari A., Bawadi H., Thompson J., Khalooei Fard R., Clark C., Ryan P.M., Ajami M., Rahimi Sakak F., Salehisahlabadi A. (2020). Relationship between body mass index, risk of venous thromboembolism and pulmonary embolism: A systematic review and dose-response meta-analysis of cohort studies among four million participants. Thromb. Res..

[24] Severinsen M.T., Kristensen S.R., Johnsen S.P., Dethlefsen C., Tjønneland A., Overvad K. (2009). Anthropometry, body fat, and venous thromboembolism: A Danish follow-up study. Circulation.

[25] Eichinger S., Hron G., Bialonczyk C., Hirschl M., Minar E., Wagner O., Heinze G., Kyrle P.A. (2008). Overweight, obesity, and the risk of recurrent venous thromboembolism. Arch. Intern. Med..

[26] Ntinopoulou P., Ntinopoulou E., Papathanasiou I.V., Fradelos E.C., Kotsiou O., Roussas N., Raptis D.G., Gourgoulianis K.I., Malli F. (2022). Obesity as a Risk Factor for Venous Thromboembolism Recurrence: A Systematic Review. Medicina.

[27] Zawadzka P.S., Imiela A.M., Pruszczyk P. (2025). The Interplay Between Obesity and Venous Thromboembolism: From Molecular Aspects to Clinical Issue. Int. J. Mol. Sci..

[28] Santoyo Villalba J., Arcelus J.I., Expósito-Ruiz M., Martínez de Mandojana A., Soler Simon S., García Ortega A., Catella J., Villares Fernández P., Ortega-Paz L., Monreal M. (2025). Influence of patient’s weight and body mass index on presentation, time of onset, and impact of postoperative venous thromboembolism (RIETE registry). J. Thromb. Thrombolysis.

[29] Mertens I., Van Gaal L.F. (2002). Obesity, haemostasis and the fibrinolytic system. Obes. Rev..

[30] Młynarska E., Bojdo K., Frankenstein H., Krawiranda K., Kustosik N., Lisińska W., Rysz J., Franczyk B. (2025). Endothelial Dysfunction as the Common Pathway Linking Obesity, Hypertension and Atherosclerosis. Int. J. Mol. Sci..

[31] Caturano A., Rocco M., Tagliaferri G., Piacevole A., Nilo D., Di Lorenzo G., Iadicicco I., Donnarumma M., Galiero R., Acierno C. (2025). Oxidative Stress and Cardiovascular Complications in Type 2 Diabetes: From Pathophysiology to Lifestyle Modifications. Antioxidants.

[32] De Pergola G., Pannacciulli N. (2002). Coagulation and fibrinolysis abnormalities in obesity. J. Endocrinol. Invest..

[33] Skurk T., Hauner H. (2004). Obesity and impaired fibrinolysis: Role of adipose production of plasminogen activator inhibitor-1. Int. J. Obes. Relat. Metab. Disord..

[34] Qu J., Cheng Y., Wu W., Yuan L., Liu X. (2021). Glycocalyx Impairment in Vascular Disease: Focus on Inflammation. Front. Cell Dev. Biol..

[35] Tarbell J.M., Cancel L.M. (2016). The glycocalyx and its significance in human medicine. J. Intern. Med..

[36] Altamura S., Lombardi F., Palumbo P., Cinque B., Ferri C., Del Pinto R., Pietropaoli D. (2024). The Evolving Role of Neutrophils and Neutrophil Extracellular Traps (NETs) in Obesity and Related Diseases: Recent Insights and Advances. Int. J. Mol. Sci..

[37] Freitas D.F., Colón D.F., Silva R.L., Santos E.M., Guimarães V.H.D., Ribeiro G.H.M., de Paula A.M.B., Guimarães A.L.S., Dos Reis S.T., Cunha F.Q. (2022). Neutrophil extracellular traps (NETs) modulate inflammatory profile in obese humans and mice: Adipose tissue role on NETs levels. Mol. Biol. Rep..

[38] Bikdeli B., Madhavan M.V., Jimenez D., Chuich T., Dreyfus I., Driggin E., Nigoghossian C., Ageno W., Madjid M., Guo Y. (2020). COVID-19 and Thrombotic or Thromboembolic Disease: Implications for Prevention, Antithrombotic Therapy, and Follow-Up: JACC State-of-the-Art Review. J. Am. Coll. Cardiol..

[39] Langan E.A., Wienandt M., Bayer A., Ellebrecht L., Kahle B. (2023). Effect of obesity on venous blood flow in the lower limbs. J. Dtsch. Dermatol. Ges..

[40] Ortega M.A., Fraile-Martínez O., García-Montero C., Álvarez-Mon M.A., Chaowen C., Ruiz-Grande F., Pekarek L., Monserrat J., Asúnsolo A., García-Honduvilla N. (2021). Understanding Chronic Venous Disease: A Critical Overview of Its Pathophysiology and Medical Management. J. Clin. Med..

[41] Obi A.T., Wakefield T.W. (2021). Calf muscle pump dysfunction and VTE risk. Blood.

[42] Liu F., Tan J., Pan Y. (2025). Prevention of deep vein thrombosis in postoperative orthopedic patients: A hybrid meta-analysis and clinical case study. Front. Med..

[43] Aziz C.B., Omar N., Abdullah W.Z., Jalil R.A., Nik W.S., Zakaria R. (2014). Reduced fibrinogen, fibrinolytic biomarkers, and physical parameters after a weight-loss program in obese subjects. N. Am. J. Med. Sci..

[44] Lassere M.N. (2008). The Biomarker-Surrogacy Evaluation Schema: A review of the biomarker-surrogate literature and a proposal for a criterion-based, quantitative, multidimensional hierarchical levels of evidence schema for evaluating the status of biomarkers as surrogate endpoints. Stat. Methods Med. Res..

[45] Campello E., Zabeo E., Radu C.M., Spiezia L., Foletto M., Prevedello L., Gavasso S., Bulato C., Vettor R., Simioni P. (2016). Dynamics of circulating microparticles in obesity after weight loss. Intern. Emerg. Med..

[46] Carvalho L., Almeida R.F., Nora M., Guimarães M. (2023). Thromboembolic Complications After Bariatric Surgery: Is the High Risk Real?. Cureus.

[47] Harrington L.B., Benz L., Haneuse S., Johnson E., Coleman K.J., Courcoulas A.P., Li R.A., Theis M.K., Cooper J., Chin P.L. (2024). Bariatric Surgery and the Long-Term Risk of Venous Thromboembolism: A Population-Based Cohort Study. Obes. Surg..

[48] Leslie Z.D., Quinn C.M., Ikramuddin S., Wise E.S. (2025). Venous Thromboembolism After Bariatric Surgery: An Analysis of Predictors and Trends in Two Large Datasets. Am. Surg..

[49] Mechanick J.I., Youdim A., Jones D.B., Garvey W.T., Hurley D.L., McMahon M.M., Heinberg L.J., Kushner R., Adams T.D., Shikora S. (2013). Clinical practice guidelines for the perioperative nutritional, metabolic, and nonsurgical support of the bariatric surgery patient--2013 update: Cosponsored by American Association of Clinical Endocrinologists, The Obesity Society, and American Society for Metabolic & Bariatric Surgery. Obesity.

[50] Amaral F.C., Baptista-Silva J.C., Nakano L.C., Flumignan R.L. (2022). Pharmacological interventions for preventing venous thromboembolism in people undergoing bariatric surgery. Cochrane Database Syst. Rev..

[51] Kröll D., Nett P.C., Rommers N., Borbély Y., Deichsel F., Nocito A., Zehetner J., Kessler U., Fringeli Y., Alberio L. (2023). Efficacy and Safety of Rivaroxaban for Postoperative Thromboprophylaxis in Patients After Bariatric Surgery: A Randomized Clinical Trial. JAMA Netw. Open.

[52] Tyselskyi V., Tryliskyy Y., Poylin V., Kebkalo A. (2023). Prolonged thromboprophylaxis with rivaroxaban after bariatric interventions: Single centre experience. Pol. Przegl Chir..

[53] Simard C., Le Gal G., Lazo-Langner A., Tagalakis V., Grewal K., Nguyen F., Wang T.F., Siegal D.M., Anvari M., Doumouras A.G. (2026). Association Between Bariatric Surgery and the Long-Term Risk for Venous Thromboembolism: A Population-Based Matched Cohort Study. Am. J. Hematol..

[54] Boccatonda A., Simion C., Brighenti A., D’Ardes D., Piscaglia F., Serra C., Simioni P., Campello E. (2026). Venous thromboembolism with modern glucose-lowering agents in diabetes: Active-comparator evidence beyond placebo-based meta-analyses. Intern. Emerg. Med..

[55] Caruso I., Cignarelli A., Sorice G.P., Perrini S., Giorgino F. (2024). Incretin-based therapies for the treatment of obesity-related diseases. npj Metab. Health Dis..

[56] Lee A.A., Khotina V.A., Kashirskikh D.A., Voronko O.E., Gasanov V.A.O., Vasiliev A.V. (2025). Incretin-Based Therapies Through the Decades: Molecular Innovations and Clinical Impact. Med. Sci..

[57] Ioannides-Demos L.L., Piccenna L., McNeil J.J. (2011). Pharmacotherapies for obesity: Past, current, and future therapies. J. Obes..

[58] Yamashita A., Asada Y. (2023). Underlying mechanisms of thrombus formation/growth in atherothrombosis and deep vein thrombosis. Pathol. Int..

[59] Libby P., Buring J.E., Badimon L., Hansson G.K., Deanfield J., Bittencourt M.S., Tokgözoğlu L., Lewis E.F. (2019). Atherosclerosis. Nat. Rev. Dis. Primers.

[60] Galli M., Benenati S., Laudani C., Simeone B., Sarto G., Ortega-Paz L., Rocco E., Bernardi M., Spadafora L., D’Amario D. (2025). Cardiovascular Effects and Tolerability of GLP-1 Receptor Agonists: A Systematic Review and Meta-Analysis of 99,599 Patients. J. Am. Coll. Cardiol..

[61] Witkowski M., Landmesser U., Rauch U. (2016). Tissue factor as a link between inflammation and coagulation. Trends Cardiovasc. Med..

[62] Ryan T.A.J., Preston R.J.S., O’Neill L.A.J. (2022). Immunothrombosis and the molecular control of tissue factor by pyroptosis: Prospects for new anticoagulants. Biochem. J..

[63] Prandoni P., Bilora F., Marchiori A., Bernardi E., Petrobelli F., Lensing A.W., Prins M.H., Girolami A. (2003). An association between atherosclerosis and venous thrombosis. N. Engl. J. Med..

[64] Cahill K.N., Amin T., Boutaud O., Printz R., Newcomb D.C., Foer D., Hodson D.J., Broichhagen J., Beckman J.A., Yu C. (2022). Glucagon-Like Peptide-1 Receptor Regulates Thromboxane-Induced Human Platelet Activation. JACC Basic. Transl. Sci..

[65] Bendotti G., Montefusco L., Lunati M.E., Usuelli V., Pastore I., Lazzaroni E., Assi E., Seelam A.J., El Essawy B., Jang J. (2022). The anti-inflammatory and immunological properties of GLP-1 Receptor Agonists. Pharmacol. Res..

[66] Ngabea M.A., Dimeji I.Y. (2025). GLP-1 receptor agonists and inflammatory pathway modulation: Dual targeting of metabolic and immune dysfunction in insulin resistance. Biochem. Biophys. Res. Commun..

[67] Cameron-Vendrig A., Reheman A., Siraj M.A., Xu X.R., Wang Y., Lei X., Afroze T., Shikatani E., El-Mounayri O., Noyan H. (2016). Glucagon-Like Peptide 1 Receptor Activation Attenuates Platelet Aggregation and Thrombosis. Diabetes.

[68] Barale C., Buracco S., Cavalot F., Frascaroli C., Guerrasio A., Russo I. (2017). Glucagon-like peptide 1-related peptides increase nitric oxide effects to reduce platelet activation. Thromb. Haemost..

[69] Simeone P., Liani R., Tripaldi R., Di Castelnuovo A., Guagnano M.T., Tartaro A., Bonadonna R.C., Federico V., Cipollone F., Consoli A. (2018). Thromboxane-Dependent Platelet Activation in Obese Subjects with Prediabetes or Early Type 2 Diabetes: Effects of Liraglutide- or Lifestyle Changes-Induced Weight Loss. Nutrients.

[70] Loganathan J., Cohen A.C., Kaloupis G.M., Harris C., Chronopoulos A., James V., Hamilton J., Green S., Wallis A., Morgan S. (2022). A pilot clinical study to Evaluate Liraglutide-mediated Anti-platelet activity in patients with type-2 Diabetes (ELAID study). J. Diabetes Complicat..

[71] Barale C., Frascaroli C., Cavalot F., Russo I. (2019). Hypercholesterolemia impairs the Glucagon-like peptide 1 action on platelets: Effects of a lipid-lowering treatment with simvastatin. Thromb. Res..

[72] Yin D.G., Ding L.L., Zhou H.R., Qiu M., Duan X.Y. (2021). Comprehensive analysis of the safety of semaglutide in type 2 diabetes: A meta-analysis of the SUSTAIN and PIONEER trials. Endocr. J..

[73] Liu Y., He L., Han S., Ping F., Li W., Xu L., Zhang H., Li Y. (2025). Glucagon-Like Peptide-1 Receptor Agonists and Risk of Venous Thromboembolism: A Systematic Review and Meta-Analysis of Randomized Controlled Trials. J. Am. Heart Assoc..

[74] Chiang C.H., Chang Y.C., Yu C.C., See X.Y., Wang T.H., Xanthavanij N., Song J., Lo S.W., Chiang C.H., Lake L. (2025). Glucagon-like peptide 1 receptor agonists and risk of venous thromboembolism: A systematic review and meta-analysis of randomized controlled trials. J. Thromb. Haemost..

[75] Chiang C.H., Song J., Chang Y.C., Osataphan S., Lee Y.C., Chang K.Y., Chi K.Y., Chiang C.H., Dua A., Lake L. (2025). Glucagon-like peptide 1 receptor agonists and venous thromboembolism in type 2 diabetes: A target trial emulation. Blood Adv..

[76] Dai H., Lee Y.A., Natalie A., Jackson W., Pham A., Levine J., Radwan R.M., Guo J., Bian J., Sheer A.J. (2026). Glucagon-Like Peptide-1 Receptor Agonists and Cardiovascular Events in Adults With Obesity and Autoimmune Disease: A Target Trial Emulation. J. Am. Heart Assoc..

[77] Micalo L., Archie M., Rowe V.L., Adewale A.P., Pham N.V., Li A., Jimenez J.C. (2026). Glucagon-like peptide-1 receptor agonists are associated with fewer venous thromboembolic events and limb complications in obese patients with chronic venous insufficiency. J. Vasc. Surg. Venous Lymphat. Disord..

[78] Mahadevan A., Vosooghi A., Arora J.S., Kumar R.S., Singh G., Tsai K.K., Quandt Z. (2026). GLP-1 receptor agonists in patients with cancer are associated with reduced all-cause mortality and hospitalization. J. Clin. Endocrinol. Metab..

[79] Kim B.I., LaValva S.M., Parks M.L., Sculco P.K., Della Valle A.G., Lee G.C. (2025). Glucagon-Like Peptide-1 Receptor Agonists Decrease Medical and Surgical Complications in Morbidly Obese Patients Undergoing Primary TKA. J. Bone Jt. Surg. Am..

[80] Marzook F.A., Kesaria A.A., Glover J.M., Alijanipour P. (2026). Preoperative Use of GLP-1 Receptor Agonists Is Associated With Reduced Risk of Postoperative DVT and Mortality in Patients Undergoing Lumbar Laminectomy. Clin. Spine Surg..

[81] Zhang J.Z., Khong J., Suresh R., Park K.E., Nguyen J.D., Zamore Z.H., Chen A.J., Elhelali A., Hultman C.S., Tuffaha S.H. (2026). GLP-1 receptor agonist use and surgical outcomes following diabetic foot reconstruction: A propensity score-matched retrospective cohort study. J. Plast. Reconstr. Aesthet. Surg..

[82] Heo K.Y., Barchick S.R., Sowa A.M., Chao M., Rajan P.V., Goh B.C., Yoon S.T. (2026). Glucagon-like peptide-1 receptor agonist use and perioperative outcomes after anterior cervical discectomy and fusion: A propensity-matched cohort study. Spine J..

[83] Amen T.B., Ibrahim L.I., Gillinov S.M., Torabian K.A., Dean M.C., Liimakka A., Lee S.K. (2025). Glucagon-like peptide-1 Agonists and Common Hand Procedures: Perioperative and Postoperative Risks and Complications. J. Hand Surg. Am..

[84] Wu J.Y., Lee K.W., Huang S.C., Chang H.Y., Lin Y.M. (2026). Comparative effectiveness of tirzepatide versus GLP-1 receptor agonists on the risk of venous thromboembolism in patients with obesity: A real-world cohort study. Front. Med..

[85] Kahkedjian F., Shah J., Kreidieh F. (2026). Tirzepatide and reduced risk of pulmonary embolism and deep vein thrombosis: A multicenter U.S. cohort study. Front. Endocrinol..

[86] Wilson B.E., Booth C.M. (2024). Real-world data: Bridging the gap between clinical trials and practice. eClinicalMedicine.

[87] Suissa S. (2008). Immortal time bias in pharmaco-epidemiology. Am. J. Epidemiol..

[88] Benchimol E.I., Smeeth L., Guttmann A., Harron K., Moher D., Petersen I., Sørensen H.T., von Elm E., Langan S.M. (2015). The REporting of studies Conducted using Observational Routinely-collected health Data (RECORD) statement. PLoS Med..

[89] Samaranayake C.B., Chen Y.C., Fang M., Rhodes C.J., Song S., Sabrin F., Ashek A., Bonnici K., Mukherjee B., Howard L.S. (2026). GLP-1 agonist, semaglutide use in acute pulmonary embolism recovery: A four-week proof-of-concept study including proteomic profiling. Eur. Heart J. Open.

[90] Kamel K.S., Chan D., Hovelroud R. (2026). Glucagon-like peptide-1 receptor agonists and venous thromboembolism: An unresolved question for Australian prescribers. Intern. Med. J..

[91] Nanah M.H., Chatterjee A., Wehbe H., Regueiro M. (2026). Comparison of IBD-related outcomes in patients with obesity treated with GLP-1 receptor agonists versus bariatric surgery. Crohns Colitis 360.

[92] Mansory E.M., Radhwi O.O. (2026). Pharmacovigilance Assessment of Thrombotic Adverse Events Linked to GLP-1 Receptor Agonists: Analysis of FAERS Reports from 2020–2025. Curr. Drug Saf..

[93] Becattini C., Cimini L.A. (2023). Provoked vs minimally provoked vs unprovoked VTE: Does it matter?. Hematol. Am. Soc. Hematol. Educ. Program..

[94] Berardicurti A., D’Andrea S., Cipollone F., Berardicurti O., Schiavone C., Boccatonda A. (2026). Effects of glucagon-like peptide-1 receptor agonists and dual glucagon-like peptide-1 receptor agonists/glucose-dependent insulinotropic polypeptide on liver stiffness and steatosis evaluated through Fibroscan^®^: A systematic review and meta-analysis. Intern. Emerg. Med..

[95] Howell R.S., Liu H.H., Brathwaite B.M., Petrone P., Akerman M., Brathwaite C.E.M. (2024). Bariatric and general surgical procedures in obese patients with a history of venous thromboembolism and concurrent anticoagulation therapy. Cir. Cir..

[96] Martin K.A., Beyer-Westendorf J., Davidson B.L., Huisman M.V., Sandset P.M., Moll S. (2021). Use of direct oral anticoagulants in patients with obesity for treatment and prevention of venous thromboembolism: Updated communication from the ISTH SSC Subcommittee on Control of Anticoagulation. J. Thromb. Haemost..

[97] Katel A., Niraula S., Aryal M., Neupane N., Neupane A., Bhatt V.R. (2025). Safety and Efficacy of Direct Oral Anticoagulants Compared to Warfarin in Patients with Venous Thromboembolism and Morbid Obesity. Int. J. Hematol. Oncol. Stem Cell Res..

[98] Shi J., Zhang Y., Tang Y., Wang L., Xiao H. (2026). Safety and efficacy of direct acting oral anticoagulants in the treatment of patients with morbid obesity with venous thromboembolism: A meta-analysis. Medicine.

[99] Jaspers T.C.C., Remmelzwaal P.C., Weersink E.P.S., Meijer K., Khorsand N. (2025). A systematic review on anti-Xa monitoring in the therapeutic use of low-molecular-weight heparins. J. Thromb. Haemost..

[100] Zhu S., Ni Q., He J. (2026). Revisiting low-molecular-weight heparin for venous thromboembolism: From pharmacology to precision dosing and implementation. Front. Pharmacol..

[101] Kahn S.R., Shrier I., Kearon C. (2008). Physical activity in patients with deep venous thrombosis: A systematic review. Thromb. Res..

[102] Gurumurthy G., Thachil J. (2026). Nonanticoagulant Strategies for Venous Thromboembolism Prophylaxis. Semin. Thromb. Hemost..

